# Does Dry Eye Affect Repeatability of Corneal Topography Measurements?

**DOI:** 10.4274/tjo.10179

**Published:** 2018-04-25

**Authors:** Aysun Şanal Doğan, Canan Gürdal, Mehmet Talay Köylü

**Affiliations:** 1 University of Health Sciences, Dışkapı Yıldırım Beyazıt Training and Research Hospital, Ophthalmology Clinic, Ankara, Turkey

**Keywords:** Dry eye, corneal topography, repeatability

## Abstract

**Objectives:**

The purpose of this study was to assess the repeatability of corneal topography measurements in dry eye patients and healthy controls.

**Materials and Methods:**

Participants underwent consecutive corneal topography measurements (Sirius; Costruzione Strumenti Oftalmici, Florence, Italy). Two images with acquisition quality higher than 90% were accepted. The following parameters were evaluated: minimum and central corneal thickness, aqueous depth, apex curvature, anterior chamber volume, horizontal anterior chamber diameter, iridocorneal angle, cornea volume, and average simulated keratometry. Repeatability was assessed by calculating intra-class correlation coefficient.

**Results:**

Thirty-three patients with dry eye syndrome and 40 healthy controls were enrolled to the study. The groups were similar in terms of age (39 [18-65] vs. 30.5 [18-65] years, p=0.198) and gender (M/F: 4/29 vs. 8/32, p=0.366). Intra-class correlation coefficients among all topography parameters within both groups showed excellent repeatability (>0.90).

**Conclusion:**

The anterior segment measurements provided by the Sirius corneal topography system were highly repeatable for dry eye patients and are sufficiently reliable for clinical practice and research.

## Introduction

The need for precise anterior segment measurements has driven the innovation of new, more reliable devices. An ophthalmic device must provide excellent correlation among repeated measurements in order to obtain consistent values. With the advent of new diagnostic and treatment modalities for many ocular conditions, precise analysis of the anterior segment is becoming increasingly important in various contexts including intraocular lens calculations, keratoconus, cataract and refractive surgery, corneal surgery, fitting of contact lenses, and glaucoma. Corneal topography enables fast and precise measurements of the anterior segment.^1^ The Sirius topography device (Sirius; Costruzione Strumenti Oftalmici, Florence, Italy) is a dual combined Scheimpflug camera and Placido-disk topography device that provides rapid, precise measurements of a wide range of anterior segment parameters.^1^ Recent studies have proved that Sirius and similar corneal topography systems are repeatable for healthy subjects.^1,2,3,4,5^

Dry eye is a multifactorial disease of the tears and ocular surface that is accompanied by increased osmolarity of the tear film and causes symptoms of discomfort, visual disturbance, and tear film instability with potential damage to the ocular surface.^6^ The tear film provides a smooth refracting surface for the cornea.^7^ When the tear film is disrupted, the optical surface becomes irregular and may cause additional aberrations or unpredictable keratometry measurements.^8,9,10,11^ Dry eye is one of the most common entities in ophthalmology practice; previous studies have reported its prevalence as ranging between 3.9% and 16.7%, with higher rates in the elderly and women.^12,13^ As dry eye and other eye pathologies may coexist, it is essential to know the repeatability of topography measurements among dry eye patients.

The aim of this study was to assess the repeatability of measurements provided by the Sirius topography device in dry eye patients and to evaluate whether there was a difference in measurements between dry eye patients and healthy controls.

## Materials and Methods

This retrospective study was performed at a tertiary referral center was approved by the ethics committee (16.01.17 34/12), and followed the tenets of the Declaration of Helsinki.

**Figure 1:**
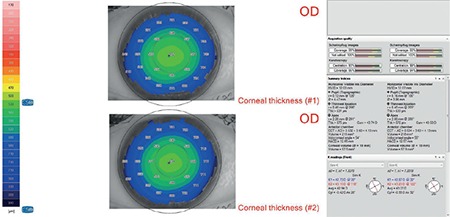
Two corneal topography images and parameters of the same eye OD: Right eye

All patients underwent ophthalmologic examination including Scheimpflug-based corneal topography (Sirius; Costruzione Strumenti Oftalmici, Florence, Italy) (Figure 1). Noninvasive break-up time (NIBUT) and meibography were also examined using the same device. 

The dry eye group constituted patients who were diagnosed according to the 2007 Report of the Definition and Classification Subcommittee of the International Dry Eye WorkShop.^6^ NIBUT values of 10 seconds or less were included in the dry eye group. The healthy control group included volunteers who had no known ophthalmic pathologies and had NIBUT longer than 10 seconds. Exclusion criteria were history of ocular disease, contact lens use, previous ocular surgery, and other anterior segment abnormalities. Patients with dry eye severity level 4 (severe and/or disabling constant discomfort and visual symptoms, marked conjunctival injection, filamentary keratitis, mucus clumping, tear debris, ulceration, trichiasis, keratinization, symblepharon) were also excluded.^6^

### Corneal Topography

The Sirius system is a topography device that combines a monochromatic 360-degree rotating Scheimpflug camera and a Placido disk to analyze the anterior segment by dual acquisition of 25 radial sections of the cornea and anterior chamber in just a few seconds. In a single scan, it provides tangential and axial curvature data of the anterior and posterior corneal surfaces, the global refractive power of the cornea, a biometric estimation of various structures, complete corneal pachymetry, and wavefront analysis. A 475 nm blue LED light is used to measure 35,632 points for the anterior corneal surface and 30,000 for the posterior corneal surface. A pachymetric map is then reconstructed using the point-by-point anterior and posterior corneal surface data. In our study, minimum corneal thickness, central corneal thickness (CCT), aqueous depth (AD), apex curvature, anterior chamber volume, horizontal anterior chamber diameter, iridocorneal angle, cornea volume, and average simulated keratometry (SimKAvg; arithmetic average of the steep and flat axes) were used for analysis. 

Topography was performed with at least >90% acquisition quality by the same experienced examiner (A.S.D.). For each eye, the corneal topography measurement was performed consecutively. All examinations were made in the same environment with stable humidity and temperature. Patients were instructed to completely refrain from using their artificial tear supplements from the previous evening until after examination. Subjects were positioned with a headrest and instructed to fixate on an internal fixation point. The patients were told to blink several times before corneal topographic images were captured. The corneal images were captured within 1-3 seconds immediately after blinking.

### Statistical Analysis

Statistical tests were performed using the Statistical Package for Social Sciences, version 20.0 (SPSS Inc, Chicago, Illinois, USA). Only the right eyes’ parameters were used for statistical assessment. In descriptive statistics, discontinuous data were shown as numbers and percentage (%); continuous data were shown as mean ± standard deviation and median (minimum-maximum). Chi-square test was used for categorical values. When comparing two groups, Student’s t test was used for continuous variables that showed normal distribution; Mann-Whitney U test was used for continuous variables that did not show normal distribution. Reliability assessment between two measurements of the same participant (intra-class correlation, [ICC]) was assessed. The ICC is an ANOVA-based correlation that measures relative homogeneity within groups between the repeated measurements in proportion to the total variation. The ICC will approach 1.0 when there is no variance within repeated measurements, indicating that total variation in measurements is due solely to variability in the parameter being measured. ICC values are commonly classified as follows: ICC <0.75, poor agreement; 0.75≤ICC<0.90, moderate agreement; ICC ≥0.90, high agreement.^14^ Parameters were also compared between the dry eye group and healthy control group. A p value less than 0.05 was considered statistically significant.

## Results

The study included 33 eyes of 33 patients with dry eye and 40 eyes of 40 healthy controls. The groups were similar in age and gender distribution, while meibomian gland loss was higher and NIBUT was lower in the dry eye group compared to healthy controls (Table 1). 

Repeated corneal topographic measurements of the same patients within both groups showed excellent agreement for minimum corneal thickness, CCT, AD, apex curvature, anterior chamber volume, horizontal anterior chamber diameter, iridocorneal angle, cornea volume, and SimKAvg. All ICCs were greater than 0.90 (Table 2).

## Discussion

Our study showed that in both the dry eye group and healthy controls, repeated dual corneal topographic measurements were in excellent agreement for minimum corneal thickness, CCT, AD, apex curvature, anterior chamber volume, horizontal anterior chamber diameter, iridocorneal angle, cornea volume, and SimKAvg. All ICCs were greater than 0.90. Especially parameters that were used in biometric measurements, CCT, SimKAvg and AD, had ICC values greater than 0.99 in dry eye except AD, which also showed a high level of agreement. One can expect that the unstable tear film may result in an irregular surface and may disrupt the topographic measurements. With tear-film instability, the quality of the refractive surface is unpredictable, often changing dramatically between blinks.^11^ In our study, we think that the speed of the measurements, obtained within a few seconds after a blink and before the tear break-up time, increased ICC. In addition, the high quality images, careful and skillful data image acquisition by the same user, and constant room conditions might have role in high repeatability results. The meaning of relatively lower ICC may be as a result of different NIBUT patterns (e.g., central vs. peripheral dry spots); however, this study did not include dry spot patterns. 

The quality of images is automatically determined by the corneal topographer. In our study, two images with high quality were included in order to avoid the risk of operator bias. Our results suggest that, particularly in dry eye patients, repeated numerous measurements may be more time consuming but are required to achieve images with the highest quality. This can be an objective for further investigation.

To the best of our knowledge, the effect of dry eye on the repeatability of anterior segment topography has not been reported to date except for CCT. Lee et al.^15^ reported an ICC of 0.891 for CCT, which was lower than in our study, but the image quality of the accepted measurements was not mentioned. However, Epitropoulos et al.^11^ showed that intraocular lens Master device resulted in intraocular lens power calculation difference of more than 0.5 D in 10% of hyperosmolar eyes. In their study, tear hyperosmolarity was found to be associated with lower repeatability of keratometry measurements.^11^ Artificial eye drops therapy in dry eye patients has been shown to be effective in increasing corneal optical quality by improving the higher order aberrations^8^ and topographic ectasia parameters^16^ of the anterior corneal surface. Zemova et al.^17^ showed that there was no association between dry eye and topographic changes in keratoconus patients by Pentacam topography device. Koh et al.^7^ reported that ocular forward light scattering and corneal backward light scattering from the anterior cornea were greater in dry eyes than in normal eyes using the C-Quant straylight meter of Oculus Scheimpflug imaging system. Akyol Salman et al.^18^ reported that there was no significant difference between dry eye patients and healthy controls in terms of ultrasonic pachymetry measurements. However, in the study of Dayanir et al.^19^, preventing the patients from blinking during ultrasonic pachymetry measurement caused a significant decrease in corneal thickness during 1 minute of drying.

There are several reports regarding the repeatability of Sirius Scheimpflug-Placido topography devices in the current literature which are consistent with the results of our study. These studies were conducted in healthy eyes, in eyes after myopic refractive surgery, and in eyes with keratoconus.^1,3,4,20^ To the best of our knowledge, there have been no repeatability studies in dry eye patients conducted with corneal topography devices. In our dry eye group, which excluded severe cases, the repeatability of corneal topography parameters was excellent.

Other anterior segment imaging systems have also been shown to have good repeatability. Güler et al.^21^ assessed the repeatability and reproducibility of the anterior segment measurements performed with a Galilei dual Scheimpflug analyzer in normal, keratoconic, and post-refractive surgery corneas and showed that the measurements showed good repeatability and reproducibility. Martin et al.^22^ assessed the repeatability of corneal thickness in healthy eyes with the Pentacam instrument and showed good repeatability. Ortiz-Toquero et al.^23^ compared the repeatability of Oculus Keratograph in a sample of healthy and keratoconus eyes and found repeatable measurements in both groups.

### Study Limitations

The present study has several limitations. In our sample, severity level 4 cases were not included. Further studies in a larger group including severe cases with high tear film osmolarity may be required in order to validate the diagnostic ability of this device in all dry eye patients.

## Conclusion

In conclusion, the Scheimpflug imaging-based Sirius corneal topography system provides repeatable examinations in both dry eyes and healthy eyes. Therefore, besides the healthy population, the device may be useful even in dry eye syndrome as a screening and diagnostic tool for anterior segment pathologies. Further studies are needed to compare the repeatability of measurements in very severe dry eye patients.

## Figures and Tables

**Table 1:**
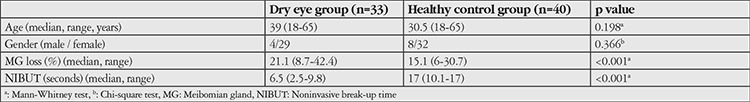
The groups were similar in terms of age and gender (p>0.05). The dry eye group had significantly higher meibomian gland loss and lower noninvasive break-up time values

**Table 2:**
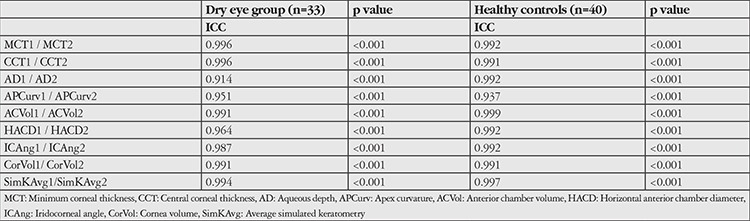
Intra-class correlations were excellent for comparison of two images in terms of all parameters within both the dry eye group and healthy controls
